# Association Between Obesity and Intra-Abdominal Solid Organ Damage in Patients with Blunt Abdominal Trauma: A Cross-Sectional Study

**DOI:** 10.3390/jcm13237467

**Published:** 2024-12-08

**Authors:** Jae Sik Chung, Sanghyun An, Hyeun Eui Moon, Yonsu Kim, Tae-Ha Chung

**Affiliations:** 1 Department of Surgery, Wonju Severance Christian Hospital, Yonsei University Wonju College of Medicine, Wonju 26426, Republic of Korea; gsjaesik@yonsei.ac.kr (J.S.C.); uldura@yonsei.ac.kr (S.A.); 2 Department of Medicine, Graduate School, Yonsei University Mirae Campus, Wonju 26493, Republic of Korea; hyuneui204@yonsei.ac.kr; 3 Research Group of Functional Medicine and Preclinical Disease, Yonsei University Wonju College of Medicine, Wonju 26426, Republic of Korea; 4 Department of Healthcare Administration and Policy, School of Public Health, University of Nevada, Las Vegas, NV 89154, USA; yonsu.kim@unlv.edu; 5 Department of Family Medicine, Wonju Severance Christian Hospital, Yonsei University Wonju College of Medicine, Wonju 26426, Republic of Korea

**Keywords:** trauma, obesity, overweight, solid organ injury

## Abstract

**Background/Objectives:** The global prevalence of obesity continues to rise. However, whether obesity affects the degree of intra-abdominal solid organ damage following blunt trauma remains unclear. This study aimed to investigate the correlation between obesity and intra-abdominal solid organ damage. **Methods:** This cross-sectional study was conducted at a regional trauma center in the Republic of Korea from January 2018 to December 2022 and included 582 patients aged 18–98 years with blunt abdominal trauma. Patients were categorized into four groups—underweight, normal weight, overweight, and obesity—based on their body mass index (BMI). Odds ratios (ORs), beta coefficients, and 95% confidence intervals (CIs) for intra-abdominal organ damage were calculated across BMI categories using multiple logistic regression analysis after adjusting for the confounding variables. **Results:** The obesity group exhibited a significant decrease in the prevalence of liver injury (OR: 0.553, CI: 0.316 to 0.966) and a reduction in liver injury severity (β: −0.214, CI: −0.391 to −0.037) compared with the normal-weight group after adjusting for the confounding factors. However, no significant association was observed between the BMI and injuries to other solid organs, such as the spleen, pancreas, and kidneys. Additionally, the younger obesity group (participants aged < 45 years) exhibited a significant negative association with both liver injury and injury grade. However, the older obesity group (participants aged > 65 years) exhibited a statistically significant association only with the liver injury grade compared with the normal-weight group. **Conclusions:** Obesity can serve as a predictive factor for the presence and severity of liver damage caused by blunt abdominal trauma.

## 1. Introduction

The global prevalence of obesity continues to rise, with approximately 39% of individuals currently affected worldwide. In South Korea, the obesity rate has also steadily increased over the past decade, from 29.7% in 2009 to 36.3% in 2019 [1,2,3], leading to an increase in the number of patients with obesity presenting with traumatic injuries as well as other medical conditions. Obesity increases the risk of various metabolic diseases such as diabetes, hypertension, and cardiovascular diseases [4,5]. However, in certain patient populations, obesity has demonstrated a protective effect on mortality, a phenomenon known as the “obesity paradox” [6,7]. Previous studies have reported a U- or J-shaped relationship between body mass index (BMI) and mortality, with overweight and patients with class I obesity exhibiting increased survival rates compared with normal-weight and underweight patients. In contrast, patients with class 2 or 3 obesity showed an increasing trend in mortality. This paradoxical association has been observed in patients with coronary heart disease, diabetes, and other critical diseases [7,8,9].

Obesity can affect the prognosis in patients with trauma [10,11]; however, the findings remain controversial. Most studies examining the relationship between obesity and trauma have focused on patient mortality or orthopedic outcomes [12,13,14]. In contrast, studies investigating whether obesity affects the degree of damage to intra-abdominal solid organs remain limited. If obesity—particularly abdominal obesity—provides a protective “cushion” effect for solid organs in cases of blunt abdominal trauma, subcutaneous or visceral fat could potentially mitigate intra-abdominal organ damage [15]. Such findings may confirm whether the obesity paradox applies to the degree of solid organ damage resulting from blunt trauma.

Therefore, this study aimed to determine the effect of obesity on the presence or grade of intra-abdominal solid organ injuries caused by blunt abdominal trauma.

## 2. Materials and Methods

### 2.1. Study Population

We retrospectively reviewed the electronic medical records (EMRs) of 648 patients with blunt abdominal trauma who presented at the trauma center of Wonju Severance Christian Hospital, a regional trauma center in the Republic of Korea, equivalent to a level 1 trauma center in the United States, between January 2018 and December 2022 [16]. The study was conducted in accordance with the ethical principles of the Declaration of Helsinki and approved by the Institutional Review Board of Yonsei University Wonju College of Medicine.

Data of the patients who visited the trauma center during the study period were obtained through an EMR review. The inclusion criteria were adult patients aged >18 years who suffered blunt abdominal trauma. The exclusion criteria included patients who did not undergo a computed tomography (CT) scan for any reason (*n* = 50) and those lost to follow-up owing to transfer to another hospital (*n* = 16). After excluding 66 participants, 582 participants aged 18–98 years were included in the final analysis.

### 2.2. Definition of Organ Injury Grade

The injury grade of intra-abdominal solid organs was assessed using the organ injury scale developed by the American Association for the Surgery of Trauma [17,18,19]. The grade of injury to the liver, spleen, pancreas, and kidney was determined based on CT images and intraoperative findings interpreted by trauma surgeons and radiologists.

### 2.3. Definitions of Obesity

The World Health Organization defines obesity as a condition of abnormal or harmful excessive fat accumulation in adipose tissue [3]. The BMI is a relatively simple method to predict body fat percentage and is calculated as follows:

BMI = Weight (kg)/Height (m^2^)

Differences exist between the standards of obesity in the West and Asia. In Asians, many studies have reported that the risk of complications of metabolic syndrome, such as hypertension and diabetes, begins to increase at a BMI of ≤25 kg/m^2^. Consequently, BMI, defining obesity, is lower in Korea than in the West [20]. In this study, obesity classes were defined using the 2020 Korean Society for the Study of Obesity treatment guidelines as follows: low weight, BMI < 18.5 kg/m^2^; normal weight, 18.5–22.9 kg/m^2^; overweight, 23–24.9 kg/m^2^; and obesity, ≥25 kg/m^2^ [21].

### 2.4. Measurements of Variables

General information about the participants was obtained through the EMR upon admission to our hospital’s trauma center. Blood pressure (BP) was measured using a noninvasive BP cuff with the patient lying in bed. A patient monitoring system (CARESCAPE Monitor B650, GE Healthcare, Helsinki, Finland) automatically monitored the pulse rate and body temperature. Blood sampling was performed immediately after admission to the trauma center for all patients. Hemoglobin (Hb) and the delta neutrophil index (DNI) were measured using an ADVIA 2120i analyzer (Siemens, Washington, DC, USA). Serum lactate was measured with the RAPIDPoint 500 analyzer (Siemens, USA), and serum aspartate aminotransferase (AST), alanine aminotransferase (ALT), amylase, lipase, blood urea nitrogen (BUN), and creatinine (Cr) were measured using the Atellica Solution analyzer (Siemens, USA). The prothrombin time/international normalized ratio was measured with the CS-5100 analyzer (Sysmex, Kobe, Japan).

### 2.5. Statistical Analysis

Continuous variables are expressed as means ± standard errors, and categorical variables are expressed as counts and percentages (%). The BMI was categorized into four groups: low weight, normal weight, overweight, and obesity. A one-way analysis of variance was used to compare the continuous variables across BMI categories by treating the group averages as continuous variables. A chi-square test was performed for the differential analysis of categorical variables based on the BMI category. Multiple logistic regression (LR) and generalized logistic regression (GLR) models were used to calculate the odds ratio (OR) for organ injury and beta coefficients for organ injury grade, respectively. In multivariate LR models, organ injury served as the independent variable, and the BMI category (continuous or categorical group) as the dependent variable. In the multivariate GLR models, the independent and dependent variables were set as the organ injury grade and BMI category, respectively. All statistical analyses were performed using the R program (version 4.1.3). Statistical significance was set at a *p* < 0.05.

## 3. Results

Table 1 presents the general characteristics of the participants according to the BMI category. Systolic BP (SBP), diastolic BP (DBP), mean arterial blood pressure (MAP), Glasgow coma scale (GCS) score, weight, lactate, Hb, DNI, AST, ALT, BUN, and hemoperitoneum prevalence exhibited positive trends. Conversely, age, PR, Cr, amylase, liver injury, pancreatic injury, and kidney injury exhibited a negative trend according to the BMI category.

**Figure 1:**
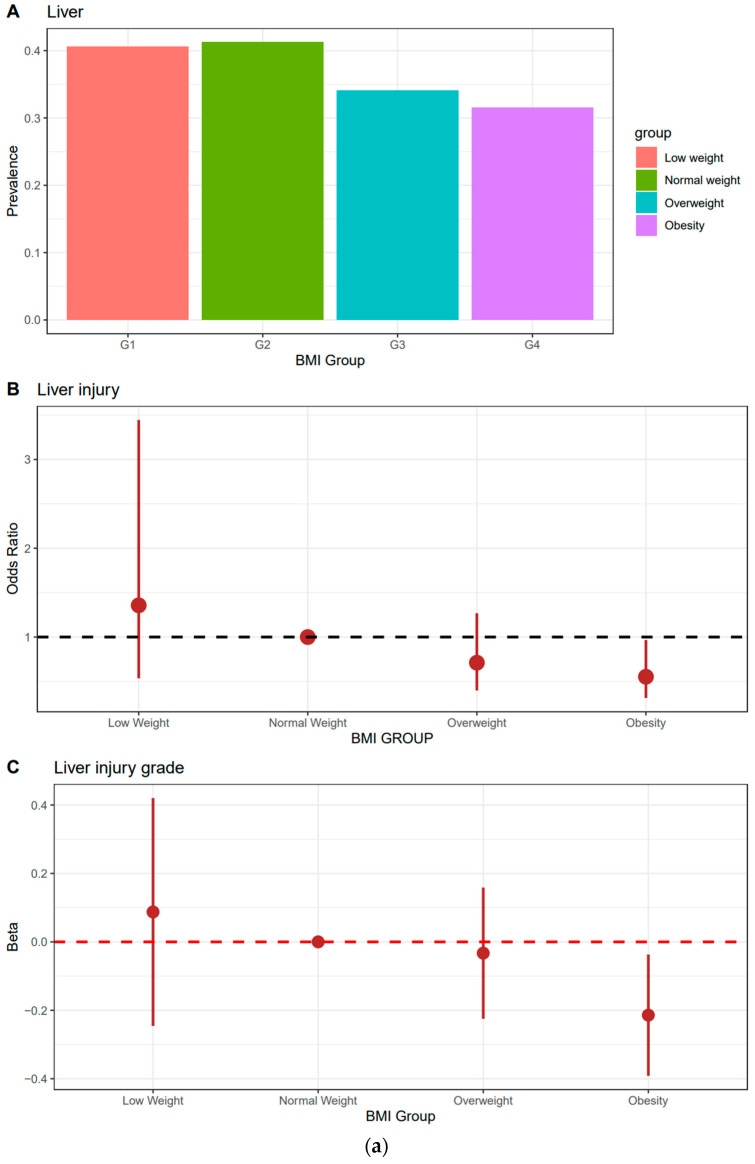

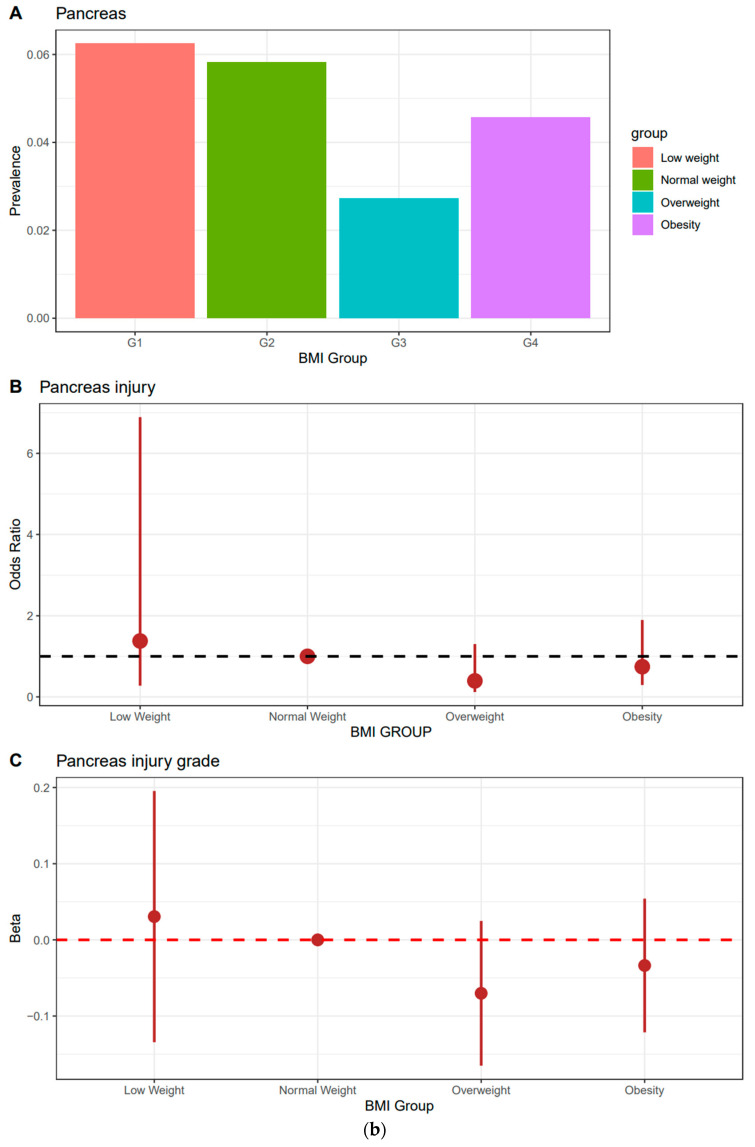

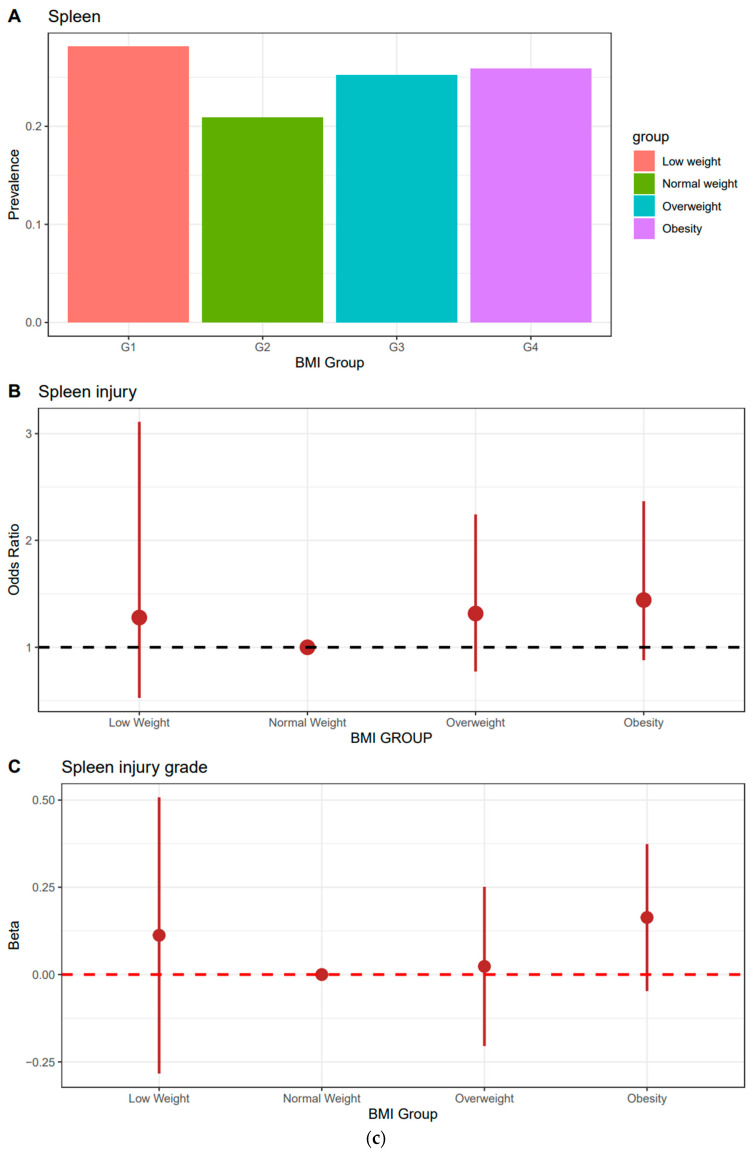

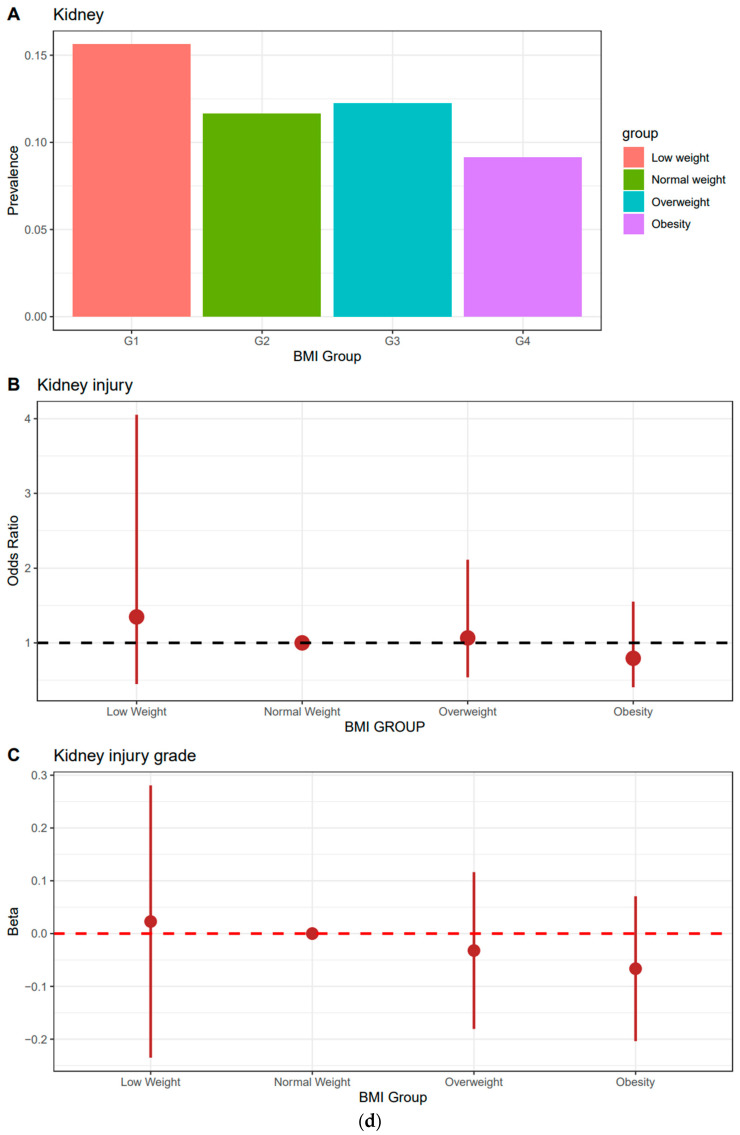
Prevalence, the odds ratio for organ injury, and the beta coefficient for organ injury grade. (**a**) Liver, (**b**) pancreas, (**c**) spleen, and (**d**) kidney. Kidney damage was defined as the presence of damage on at least one side, and the grade was analyzed based on the higher grade on either side. G1, group 1 (Low weight); G2, group 2 (Normal weight); G3, group 3 (Overweight); G4, group 4 (Obesity).

Table 2 and Figure 1 present the ORs and beta coefficients for solid organ injury and injury grade across the BMI categories, determined using multiple LR and GLR, respectively. Kidney injury was defined as damage on at least one side, with the grade determined based on the higher injury grade on either side. Adjusting for age, sex, SBP, DBP, PR, lactate, Cr, Hb, DNI, AST, and ALT, the obesity group demonstrated a significant decrease in the prevalence of liver injury (OR: 0.553, 95% CI: 0.316 to 0.966), with a negative trend in the beta coefficients for the liver injury grade (−0.214, 95% CI: −0.391 to −0.037). The overweight group exhibited a negative trend in the OR and beta coefficient for pancreatic injury (OR: 0.375, 95% CI: 0.119 to 1.303; beta coefficient: −0.070, 95% CI: −0.165 to 0.025), whereas the obesity group exhibited insignificant results (OR: 0.744, 95% CI: 0.892 to 1.897; beta coefficient: −0.033, 95% CI: −0.121 to 0.054). Kidney injury exhibited a negative trend for the OR (OR: 0.793, 95% CI: 0.405 to 1.553) and beta coefficient (−0.066, 95% CI: −0.203 to 0.071) in the obesity group, although the results were not significant. Spleen injury exhibited a U-shaped correlation with the BMI category, with the normal-weight group showing the lowest OR and beta coefficients, whereas the low-weight, overweight, and obesity groups demonstrated progressively higher values.

Table 3 shows the results of the subgroup analyses of liver injury and BMI categories according to age. Age was categorized into three groups: younger (<45 years), middle-aged (45–65 years), and older (>65 years). In the younger group, the OR for liver injury in the obesity group significantly decreased to 0.127 compared to the normal-weight group (95% CI: 0.040 to 0.402); additionally, the beta coefficient for the liver injury grade also significantly decreased by −0.667 in the obesity group (95% CI: −1.024 to −1.310). The middle-aged group exhibited insignificant results, with the OR for liver injury being 1.440 (95% CI: 0.640 to 3.238) and with the beta coefficient for injury grade being 0.017 (95% CI: −0.272 to 0.307). In contrast, the older group exhibited a significant OR of 0.208 (95% CI: 0.029 to 1.449) for liver injury and an insignificant beta coefficient of −0.250 (95% CI: −0.485 to −0.017) for the liver injury grade in the obesity group.

## 4. Discussion

Our study found that the risk of liver injury and injury grade in Korean patients with blunt trauma was significantly lower in the obesity group compared with the normal-weight group, particularly in the younger group. This group exhibited significant results for both liver injury and injury grade. In the older group, significant findings were observed only for the degree of liver injury.

Previous studies have focused on the correlation between obesity and prognosis after trauma. Brahmbhatt et al. [22] showed that trauma in morbid patients with obesity is associated with a poor prognosis, including increased mortality, multiple organ failure, and acute renal failure. Durgun et al. [23] reported that obesity increases mortality and morbidity, regardless of injury severity. Additionally, the length of hospital stay, rate and length of ICU stay, rate of extremity injury, multiple traumas, and death rate increased with an increase in the BMI of patients with trauma. Spitler et al. [24] also noted that obesity increases the risk of posttraumatic complications, including infections, multiorgan failure, and death, which may be attributed to unique physiological differences, such as higher ventilation demands in the respiratory system, a higher risk of thromboembolic events, and disruption of the immune system, that make initial resuscitation more difficult and less effective in patients with obesity than in patients with normal weight.

However, some studies have reported positive outcomes related to obesity and post-trauma prognosis. Dvorak et al. [6] reported that overweight patients have improved odds of survival following trauma. Their findings aligned with other studies in non-trauma populations, highlighting the existence of an “obesity paradox” among trauma survivors. Additionally, in patients with considerable subcutaneous fat, the “fat cushion effect” has been associated with a reduced prevalence of severe abdominal injuries in motor vehicle accidents [15]. Arbabi et al. [25] reported similar findings, supporting the “fat cushion effect”, indicating that being overweight was associated with lower injury severity and abdominal abbreviated injury scale scores compared with being underweight.

Our study confirmed a significant association between liver damage and obesity in patients with trauma, particularly in the younger age group. Similarly, we hypothesized that obesity may have a protective effect against liver damage through the cushion effect. Notably, the adipose tissue not only stores energy and performs endocrine functions but also acts as a protective padding for organs during trauma [15,26]. However, the protective effects of obesity were not significant in the other organs. This discrepancy may be explained by the anatomical structure of the liver. Several ligaments, including the coronary, triangular, falciform, round, hepatogastric, and hepatoduodenal ligaments [27,28,29], anchor the liver to the right upper quadrant of the abdominal cavity. These ligaments restrict excessive liver movement [30], and tension or rupture of these ligaments is observed in most cases of hepatic laceration [31]. Particularly, deceleration of the liver by the very stable and rigid falciform ligament results in liver rupture along the ligament and destruction of large intrahepatic vessels, resulting in massive hemorrhage into the abdominal cavity [32]. Clinically, the fat pad of the falciform ligament was thickened in patients with obesity, as shown in previous studies [33]. Therefore, the fat pad surrounding the falciform ligament may play a more substantial role in preventing traumatic ligament damage in the obese group than in the non-obese group, consistent with the findings of this study.

The predictive power of the correlation between obesity and liver damage was better in the younger age group (Table 3). This finding likely results from the characteristics of aging and obesity. As the body ages, ligaments, muscles, and tendons lose their elasticity and weaken [34]. Consequently, ligaments are more susceptible to damage in older individuals, with associated slower healing. These changes are attributed to decreased activity in the cells that maintain the ligaments [35] and may counteract the cushioning effect of the falciform ligament’s fat pad on liver damage. Overall, these changes support the notion that young obese adults have a lower risk of liver damage. In recent years, various pharmacological interventions have been explored for their efficacy in managing obesity and related metabolic disorders. For instance, liraglutide has shown promising results in improving body composition and metabolic profiles in patients with type 2 diabetes mellitus. Rondanelli et al. (2016) conducted a study demonstrating the significant effects of liraglutide on body composition, appetite regulation, and lipid profiles over a 24-week period in overweight and obese individuals [36].

The strength of our study lies in its focus as the first investigation into the association between obesity and intra-abdominal solid organ damage in a Korean population with trauma, addressing a critical gap in studies on solid organ damage. This study focused on examining whether obesity could predict the severity of intra-abdominal solid organ damage following blunt abdominal trauma. Our findings demonstrated a significant association between obesity and both the presence and severity of liver damage. However, although obesity appeared to have a positive effect on damage to other organs, it was not significant, and an opposite trend was observed in the case of the spleen. Consequently, we cannot conclude that obesity provides a cushioning effect for all intra-abdominal solid organs following blunt trauma.

This study had some limitations. First, trauma management prioritizes initial emergency resuscitation and definitive treatment for patients with multiple traumas who visit the trauma center. Therefore, we did not assess obesity using additional methods such as body composition (InBody) or abdominal circumference measurement, which may have weakened our findings. As such, our results may have limited interpretation because we only compared participants based on a BMI without distinguishing between subcutaneous and visceral fat in cases of abdominal obesity. In the future, it is necessary to differentiate between these types of abdominal fat and to examine their relationship with organ damage. Second, the retrospective and non-randomized study design may have introduced selection bias, and as this was a cross-sectional study, causality could not be established. Third, this study was conducted solely on a Korean population, limiting the generalizability of the results to other ethnic groups. Fourth, while the cross-sectional design provides valuable insights into the relationship between obesity and organ damage at a single point in time, a longitudinal approach would allow researchers to track changes in injury severity and organ damage over time. This could help establish causal relationships and better understand how obesity influences recovery and long-term outcomes following trauma. By following patients through their treatment and recovery periods, researchers could gather more comprehensive data on how varying degrees of obesity impact healing processes and complications, thus enhancing the overall robustness of the findings. Despite these limitations, our study is the first to investigate whether obesity can serve as an early predictor of the degree of intra-abdominal solid organ damage in patients with blunt abdominal trauma, potentially providing a foundation for further studies in the future.

## 5. Conclusions

This study suggests that obesity can be considered a predictive factor for the presence and severity of liver damage following blunt abdominal trauma, particularly in younger adults. However, prospective studies utilizing tools for more accurate measurement of abdominal obesity are needed to further validate and enhance the effectiveness of the present findings.

## Figures and Tables

**Table 1:** General characteristics ^a^ according to BMI category.

	Total	Low Weight (BMI < 18.5 kg/m^2^)	Normal Weight (BMI 18.5–22.9 kg/m^2^)	Overweight (BMI 23–24.9 kg/m^2^)	Obesity (BMI ≥ 25 kg/m^2^)	*p* for Trend ^b^
Sample size (*n*)	582	32	206	147	197	-
Sex (male)	409 (70.2)	14 (43.8)	124 (60.2)	120 (81.6)	151 (76.6)	<0.001
Age	53.86 ± 0.70	57.44 ± 3.75	54.57 ± 1.23	53.31 ± 1.38	52.94 ± 1.09	0.468
SBP (mmHg)	120.77 ± 1.41	109.44 ± 7.47	119.52 ± 2.28	121 ± 2.71	123.73 ± 2.47	0.150
DBP (mmHg)	70.82 ± 0.86	61.31 ± 4.15	69.12 ± 1.41	71.95 ± 1.62	73.3 ± 1.50	0.009
MAP (mmHg)	87.47 ± 1.00	77.35 ± 5.07	85.92 ± 1.62	88.30 ± 1.90	90.11 ± 1.76	0.029
PR	87.78 ± 0.89	90.72 ± 3.37	85.87 ± 1.46	89.22 ± 1.78	88.22 ± 1.59	0.395
BT (°C)	36.54 ± 0.03	36.56 ± 0.14	36.53 ± 0.05	36.55 ± 0.05	36.54 ± 0.05	0.996
GCS	13.92 ± 0.11	13.06 ± 0.49	13.92 ± 0.18	13.89 ± 0.22	14.08 ± 0.18	0.236
Height (m)	1.66 ± 0.01	1.62 ± 0.01	1.65 ± 0.01	1.68 ± 0.01	1.67 ± 0.01	<0.001
Weight (Kg)	66.46 ± 0.57	46.00 ± 0.79	57.72 ± 0.49	68.08 ± 0.56	77.73 ± 0.97	<0.001
BMI (Kg/m^2^)	23.96 ± 0.17	17.48 ± 0.17	21.12 ± 0.09	24.00 ± 0.05	27.96 ± 0.29	<0.001
Lactate (mmol/L)	3.00 ± 0.09	2.97 ± 0.35	2.88 ± 0.15	3.04 ± 0.18	3.10 ± 0.18	0.803
Hb (g/dL)	12.88 ± 0.09	11.73 ± 0.41	12.45 ± 0.16	13.27 ± 0.16	13.24 ± 0.16	<0.001
DNI	1.56 ± 0.12	1.11 ± 0.41	1.55 ± 0.17	1.54 ± 0.17	1.68 ± 0.26	0.758
AST (U/L)	194.18 ± 11.87	167.06 ± 34.32	204.62 ± 23.21	186.95 ± 19.67	193.05 ± 19.93	0.884
ALT (U/L)	139.47 ± 8.91	108.31 ± 32.3	140.94 ± 16.45	131.04 ± 14.9	149.28 ± 15.74	0.726
BUN (mg/dL)	17.92 ± 0.29	17.17 ± 1.44	17.73 ± 0.54	18.23 ± 0.46	18.00 ± 0.5	0.843
Creatinine (mg/dL)	1.07 ± 0.03	1.23 ± 0.24	1.03 ± 0.06	1.08 ± 0.05	1.06 ± 0.03	0.535
PT/INR	1.15 ± 0.04	1.10 ± 0.03	1.23 ± 0.09	1.06 ± 0.01	1.13 ± 0.07	0.405
Amylase (U/L)	87.30 ± 10.22	141.66 ± 50.3	92.97 ± 8.1	77.95 ± 3.79	79.53 ± 4.08	0.009
Lipase (U/L)	142.57 ± 18.43	198.22 ± 89.11	125.65 ± 15.16	131.98 ± 17.08	159.15 ± 33.33	0.577
Hemoperitonium (%)	279 (47.8)	15 (46.9)	96 (46.6)	71 (48.3)	107 (54.3)	0.443
Liver injury (%)	210 (36.0)	13 (40.6)	85 (41.3)	50 (34)	62 (31.5)	0.189
Spleen injury (%)	140 (24.1)	9 (28.1)	43 (20.9)	37 (25.2)	51 (25.9)	0.595
Pancreas injury (%)	27 (4.6)	2 (6.2)	12 (5.8)	4 (2.7)	9 (4.6)	0.559
Kidney injury (%)	65 (11.2)	5 (15.6)	24 (11.7)	18 (12.2)	18 (9.1)	0.641
Mortality (%)	14 (2.4)	3 (9.4)	4 (1.9)	1 (0.7)	6 (3)	0.029

^a^ Continuous and categorical variables are described as means ± standard errors and frequencies (percentages), respectively. ^b^ *p* for trend was calculated using one-way analysis of variance or chi-square test. Abbreviations: SBP, systolic blood pressure; DBP, diastolic blood pressure; MAP, mean arterial blood pressure; PR, pulse rate; BT, body temperature; GCS, Glasgow coma scale; BMI, body mass index; Hb, hemoglobin; DNI, delta neutrophil index; AST, aspartate transaminase; ALT, alanine transferase; BUN, blood urea nitrogen; PT/INR, prothrombin time/international normalized ratio.

**Table 2:** Odds ratios for organ injury and beta coefficients for organ injury grade.

Organ Injury	Low Weight	Normal Weight	Overweight	Obesity
Sample size (*n*)	32	206	147	197
Liver	1.357 (0.535–3.445)	1 (reference)	0.711 (0.398–1.268)	0.553 (0.316–0.966) *
Spleen	1.129 (0.525–3.110)	1 (reference)	1.316 (0.772–2.243)	1.443 (0.879–2.367)
Pancreas	1.379 (0.276–6.894)	1 (reference)	0.375 (0.119–1.303)	0.744 (0.892–1.897)
Kidney	1.348 (0.448–4.053)	1 (reference)	1.067 (0.538–2.114)	0.793 (0.405–1.553)
Organ injury grade	Low weight	Normal weight	Overweight	Obesity
Sample size (*n*)	32	206	147	197
Liver	0.087 (−0.256–0.420)	0 (reference)	−0.033 (−0.225–0.159)	−0.214 (−0.391–−0.037) *
Spleen	0.112 (−0.283–0.508)	0 (reference)	0.023 (−0.205–0.251)	0.163 (−0.047–0.374)
Pancreas	0.030 (−0.134–0.196)	0 (reference)	−0.070 (−0.165–0.025)	−0.033 (−0.121–0.054)
Kidney	0.023 (−0.235–0.280)	0 (reference)	−0.032 (−0.180–0.116)	−0.066 (−0.203–0.071)

After adjusting for age, sex, SBP, DBP, PR, lactate, creatinine, Hb, DNI, AST, and ALT levels, kidney damage was defined as the presence of damage on at least one side, and the grade was analyzed based on the higher grade on either side. Definitions: low weight, BMI < 18.5 kg/m^2^; normal weight, BMI 18.5–22.9 kg/m^2^; overweight, BMI 23–24.9 kg/m^2^; and obesity, BMI ≥ 25 kg/m^2^. * *p* < 0.05. Summary statistics are described as beta coefficients or odds ratios (95% confidence interval).

**Table 3:** Subgroup analysis of liver injury and injury grade according to age group.

Liver Injury	Sample Size (*n*)	Low Weight	Normal Weight	Overweight	Obesity
Age < 45 years	171	0.443 (0.007–2.650)	1 (reference)	0.337 (0.108–1.052)	0.127 (0.040–0.402) *
Sample size (*n*)		11	56	41	63
Age 45–65 years	254	5.375 (1.124–25.693)	1 (reference)	0.602 (0.240–1.506)	1.440 (0.640–3.238)
Sample size (*n*)		9	90	67	88
Age > 65	157	2.103 (0.232–19.044)	1 (reference)	1.509 (0.371–6.133)	0.208 (0.029–1.449)
Sample size (*n*)		12	62	39	46
Liver injury grade	Sample size (*n*)	Low weight	Normal weight	Overweight	Obesity
Age < 45 years	171	0.357 (−0.248–0.960)	0 (reference)	−0.088 (−0.472–0.297)	−0.667 (−1.024–−0.310) *
Sample size (*n*)		11	56	41	63
Age 45–65 years	254	0.319 (−0.355–0.992)	0 (reference)	−0.047 (−0.360–0.265)	0.017 (−0.272–0.307)
Sample size (*n*)		9	90	67	88
Age > 65	157	0.001 (−0.376–0.379)	0 (reference)	0.034 (−0.204–0.273)	−0.250 (−0.485–−0.017) *
Sample size (*n*)		12	62	39	46

Odds ratios for liver injury and beta coefficients for liver injury grade after adjusting for age, sex, SBP, DBP, PR, lactate, creatinine, Hb, DNI, AST, and ALT. Abbreviations: low weight, BMI < 18.5 kg/m^2^; normal weight, BMI 18.5–22.9 kg/m^2^; overweight, BMI 23–24.9 kg/m^2^; and obesity, BMI ≥ 25 kg/m^2^. * denotes *p* < 0.05. Summary statistics were described as beta coefficients or odds ratios (95% confidence interval).

## Data Availability

The data presented in this study are available upon request from the corresponding author. The data were not made publicly available because of privacy concerns of the enrolled patients.
